# Linking Y‐chromosomal short tandem repeat loci to human male impulsive aggression

**DOI:** 10.1002/brb3.855

**Published:** 2017-10-16

**Authors:** Chun Yang, Huajie Ba, Yin Cao, Guoying Dong, Shuyou Zhang, Zhiqin Gao, Hanqing Zhao, Xianju Zhou

**Affiliations:** ^1^ Department of Psychiatry Psychiatry Center of Chinese People's Liberation Army No.102 Hospital of People's Liberation Army Changzhou China; ^2^ DNA Laboratory Public Security Bureau of Changzhou Changzhou China; ^3^ Department of Neurology Laboratory of Neurological Diseases Changzhou No.2 People's Hospital The Affiliated Hospital of Nanjing Medical University Changzhou China

**Keywords:** allele, behavior, genetics, haploid, haplotype, impulsive aggression, male, offender, polymorphisms, short tandem repeats, Y chromosome

## Abstract

**Introduction:**

Men are more susceptible to impulsive behavior than women. Epidemiological studies revealed that the impulsive aggressive behavior is affected by genetic factors, and the male‐specific Y chromosome plays an important role in this behavior. In this study, we investigated the association between the impulsive aggressive behavior and Y‐chromosomal short tandem repeats (Y‐STRs) loci.

**Methods:**

The collected biologic samples from 271 offenders with impulsive aggressive behavior and 492 healthy individuals without impulsive aggressive behavior were amplified by PowerPlex^R^Y23 PCR System and the resultant products were separated by electrophoresis and further genotyped. Then, comparisons in allele and haplotype frequencies of the selected 22 Y‐STRs were made in the two groups.

**Results:**

Our results showed that there were significant differences in allele frequencies at DYS448 and DYS456 between offenders and controls (*p* < .05). Univariate analysis further revealed significant frequency differences for alleles 18 and 22 at DYS448 (0.18 vs 0.27, compared to the controls, *p* = .003, OR=0.57,95% CI=0.39–0.82; 0.03 vs 0.01, compared to the controls, *p* = .003, OR=7.45, 95% CI=1.57–35.35, respectively) and for allele 17 at DYS456 (0.07 vs 0.14, compared to the controls, *p* = .006, OR=0.48, 95% CI =0.28–0.82) between two groups. Interestingly, the frequency of haploid haplotype 22‐15 on the DYS448‐DYS456 (DYS448‐DYS456‐22‐15) was significantly higher in offenders than in controls (0.033 vs 0.004, compared to the control, *p* = .001, OR = 8.42, 95%CI =1.81–39.24). Moreover, there were no significant differences in allele frequencies of other Y‐STRs loci between two groups. Furthermore, the unconditional logistic regression analysis confirmed that alleles 18 and 22 at DYS448 and allele 17 at DYS456 are associated with male impulsive aggression. However, the DYS448‐DYS456‐22‐15 is less related to impulsive aggression.

**Conclusion:**

Our results suggest a link between Y‐chromosomal allele types and male impulsive aggression.

## 1 INTRODUCTION

Human aggressive behavior is a behavior of physical, psychological, or verbal violence against other persons with hostile, threat, harmful, or destructive components. Impulsive aggressive behavior is a non‐premeditated aggressive act that an individual engages in hastily without any consideration of its consequences (Coccaro, Lee, & McCloskey, 2014; Siever, 2008). It usually occurs as an exaggerated response to emotion‐provoking events, leading to undesirable consequences. Individuals with impulsive aggressive behavior often have deficit in controlling their impulses. Most of them feel regret after their impulsive aggression. The behavior has complex biologic and social causes, and often results from the interactions of genetic and environmental factors (Chester et al., 2015). There are significant sex differences in response to stressful events. For example, men are more susceptible to impulsive behavior than women (Lovell‐Badge, 2005). Epidemiological studies revealed that the impulsive aggressive behavior is affected by genetic factors(Craig & Halton, 2009), and the male‐specific Y chromosome plays an important role in this behavior(Shah, Ayub, Firasat, Kaiser, & Mehdi, 2009). Several studies (Carey, 1992; Hesselbrock, 1991) showed that aggressive/violent behavior is associated with Y chromosome. Shah et al. (2009) found that haploid R2 and R1a1 on the human Y chromosome may be linked to the aggressive behavior. Lee and Harley (2012) proposed that male‐specific sex‐determining region on the Y chromosome (SRY), as genetic basis for sex‐related responses, has a direct relationship with men impulsive aggressive behavior and control this behavior by regulating the release of catecholamines hormones (such as noradrenaline), 5‐HT and DA. Recently, Yang et al. (2013, 2015) showed that there is an association between euchromosome STR loci (TH1 and TPOX) on the non‐Y‐chromosome and male impulsive violent behavior. However, the association between Y‐chromosomal STR loci and the male impulsive aggressive behavior has not been reported. In this study, we used PowerPlex^R^Y23 System to amplify 22STR loci (DYS576, DYS389I, DYS448, DYS389II, DYS19, DYS391, DYS481, DYS549, DYS533, DYS438, DYS437, DYS570, DYS635, DYS390, DYS439, DYS392, DYS643, DYS393, DYS458, DYS385 a/b, DYS456, and DY_GATA_H4) on the Y‐chromosome, in search of alleles and haplotypes at Y‐STR loci related to the impulsive aggressive behavior.

## 2 MATERIALS AND METHODS

### 2.1 Subjects

The criminal records of all male offenders arrested from January 2003 to December 2014 were obtained and viewed from local police stations in Changzhou, Jiangsu Province. The repeatedly aggressive behaviors (such as fighting, killing, robbing, injuring, and assaulting) of these offenders resulted in either wounding (i.e., worse than minor injury) or death to the victims. By reviewing the criminal documents, we initially screened offenders with a response to spontaneous anger‐provoking stimuli but no premeditation (regarded as impulsive violent behavior in contrast to premeditated aggression). The classification was further confirmed by the local Bureau of Public Security and well‐trained psychiatrists from our hospital, a psychiatric center of Chinese People's Liberation Army (PLA).

The inclusion criteria for this study included (i) age ranged 10–80 years; (ii) a document of impulsive violent behavior. Offenders who had any of the following conditions were also excluded from the recruitment: (i) a serious mental disorder based on the psychiatrist's diagnosis or based on a history of a mental disorder that had previously been diagnosed by a psychiatrist; (ii) a history of premeditated aggressive behavior; (iii) a history of a head injury; (iv) an obvious physical illness; or (v) a history of substance abuse problems. A total of 814 male offenders with impulsive violent behavior were initially screened by telephone. Among them, 312 did not respond, and 183 refused to participate in this study. Subsequently, the voluntary participants were interviewed by two well‐trained psychiatrists. In all, 48 offenders suffered from mental illness (16 with schizophrenia, 20 with bipolar disorders, and 12 with depression) were consistently diagnosed, and excluded in this study. Following this screening procedure, 271 male individuals with impulsive violent behavior were enrolled and signed informed consent in this study. The tools which were used for impulsive aggressive behavior involved the followings: sharp tools (216 males), blunt tools (37 males), hands (15 males), mouth bite (2 males), and 1 with firearm tool (1 male). Meanwhile, aggressive behaviors of these offenders were assessed with modified overt aggressive scale (MOAS) as described below by an experienced psychiatrist.

For the control group, age‐ and gender‐matched male individuals without impulsive aggressive behavior were recruited from male staffs of Changzhou No. 102 hospital of the People's Liberation Army (PLA), Changzhou No. 2 People's Hospital, the Public Security Bureau of Changzhou, as well as a couple of communities. Participants in the control group were screened by the same psychiatrists. Individuals with any of the following situations were excluded: (i) a history of a mental disorder in the individual or his family members; (ii) having criminal records or a history of impulsive aggressive behavior (MOAS score >0); (iii) having family members with a history of violent behavior or criminal records; and (iv) biologic relationship with any of the other individuals in the control sample. Finally, 492 eligible individuals participated in this study, and signed informed consent.

The scale for aggressive behavior MOAS is widely used to assess impulsive aggressive behavior (Kay, Wolkenfeld, & Murrill, 1988). The scale consists of four categories: (i) verbal aggression; (ii) physical aggression against objects; (iii) physical aggression against self; and (iv) physical aggression against other people. Each category is scored from 0 to 4: 0 indicates no aggressive behavior and higher scores indicate increasing severity. The total MOAS score is the sum of weighted scores for four categories. All offenders and controls were assessed for their impulsive aggressive behavior by a psychiatrist well‐trained in using the scale.

All participants in this study were Chinese males of Han ethnicity and had no kinship each other. The study was approved by the Ethics Committee for the Protection of Human Subjects of Changzhou No. 102 Hospital of PLA.

### 2.2 Sample collection

5 ml peripheral venous blood from each participant was collected in ethylene diamine tetraacetic acid (EDTA) tubes, stored at −80°C until use.

### 2.3 DNA preparation

Genomic DNA was extracted from white blood cell fractions of all samples using the chelex‐100 protocol(Walsh, Metzger, & Higushi, 2013). Specifically, added 3 μl blood sample into 0.6 ml centrifuge tube, and then added 0.5 ml sterile deionized water (dH_2_O) at room temperature for 30 min; centrifuged at 12,000r/min for 5 min, then discarded the supernatant. Repeated the above steps one time, added 150 μl 5% Chlex‐100 suspension; incubated 56°C for 30 min; vortexed and centrifuged at 12,000r/min for 5 min. Finally, the genomic DNA in the supernatant was stored at4°C for PCR.Y‐STR loci: the PowerPlex^®^ Y23 System allows co‐amplification and four‐color fluorescent detection of 22 loci, consisting of DYS576, DYS389I, DYS448, DYS389II, DYS19, DYS391, DYS481, DYS549, DYS533, DYS438, DYS437, DYS570, DYS635, DYS390, DYS439, DYS392, DYS643, DYS393, DYS458, DYS385a/b, DYS456, and Y‐GATA‐H4. The PowerPlex^®^ Y23 System provides all materials necessary to amplify Y‐STR regions of human genomic DNA, including a hot‐start DNA polymerase (Thompson et al., 2013). The haploid composed by 22 Y‐STR loci can be conservatively transmitted in the same paternal (with an exception of mutation), and the linked coding region information also will be transmitted. The related information of Y‐STR loci is seen in Table 1.

**Table 1:** The related information of 22 Y‐STR loci in the PowerPlex^®^ Y23 system

STR Loci	Location on chromosome	Core repeat (5′→3′)	Allele range	Allele repeat frequency
DYS576	Y	AAAG	97–145	11–23
DYS389I	Y	(TCTG)(TCTA)	147–179	9–17
DYS448	Y	AGAGAT	196–256	14–24
DYS389II	Y		259–303	24–35
DYS19	Y	TAGA	312–352	9–19
DYS391	Y	TCTA	86–130	5–16
DYS481	Y	CTT	139–184	17–32
DYS549	Y	GATA	198–238	7–17
DYS533	Y	ATCT	245–285	7–17
DYS438	Y	TTTTC	293–343	6–16
DYS437	Y	TCTA	344–380	11–18
DYS570	Y	TTTC	90–150	10–25
DYS635	Y	TSTA compound	150–202	15–28
DYS390	Y	(TCTA) (TCTG)	207–255	17–29
DYS439	Y	AGAT	263–307	6–17
DYS392	Y	TAT	314–362	4–20
DYS643	Y	CTTTT	368–423	6–17
DYS393	Y	AGAT	101–145	7–18
DYS458	Y	GAAA	159–215	10–24
DYS385a/b	Y	GAAA	223–307	7–28
DYS456	Y	AGAT	316–364	11–23
DY_GATA_H4	Y	TAGA	374–414	8–18

### 2.4 PCR amplification

The PowerPlex^®^ Y23 System (Promega, Wisconsin, USA), a multiplex amplification system with fluorescent detection, was used in this study. The amplification was performed following the manufacturer's instructions. Extracted DNA was amplified in a total reaction volume of 10 μl, containing 1 μl of genomic DNA template, 2 μl of PowerPlex^R^Y23 5 × Master Mix, 1 μl of PowerPlex^®^ Y23 10 × Primer Pair Mix, and 6 μl of ddH2O. The amplification was performed on a thermal cycler (Applied Biosystems GeneAmp^®^ PCR system 9700) using the following cycling parameters: 96°C for 2 min; 27 cycles of 94°C for 10 s, 61°C for 60 s, 72°C for 30 s; 60°C extension for 20 min; holding at 4°C. 2800M Control DNA was used as a positive PCR control in all amplifications. Sterile deionized water was used as a negative control in all PCR batches Detection of PCR products: All analyses utilized the CC5 Internal Lane Standard 500 Y23 (ILS) and the allelic ladder mix which were provided with the Powerplex^®^ Y23 System. Separation of amplification products was conducted on the Applied Biosystems 3500xL Genetic Analyzer series (Life Technologies^™^, Carlsbad, CA). Samples were prepared for separation and analysis by adding a mixture of 1 μl amplified product or DNA allelic ladder or dH_2_O (blank control), 0.5 μl CC5 ILS to 14 μl of 37:1 Hi‐Di^™^ formamide (Life Technologies^™^, Carlsbad, CA). A 36 cm capillary array was used on all instruments with POP‐4^R^ Polymer (Life Technologies^™^, Carlsbad, CA) under 15 kV and at 60°C. Sample data were collected using collection software V1.0 (Life Technologies^™^, Carlsbad, CA). Allele sizing and calling were performed using Gene MapperID‐X v1.3 (Life Technologies^™^, Carlsbad, CA).

### 2.5 Statistical analysis

SPSS software version 19.0 (SPSS Inc., Chicago, IL, USA) was used to perform statistical comparisons between two groups, and the significance level was set at 0.05. The PowerStats software (Promega, Wisconsin, USA) was utilized to obtain alleles, haploid haplotype frequencies of 22Y‐STR loci. R×C chi‐squared tests were used to compare the distributions of alleles, haploid haplotypes (alleles and haplotypes with lower than 1% frequency were excluded in two groups). Fisher's exact test was employed to calculate value if there were more than 20% lattice theoretical frequency (T) <5 or T<1. When R×C chi‐squared test was significant at the 0.05 level, allele or haplotype frequencies of these STR loci was subsequently compared using 2 × 2 chi‐squared tests. When T < 5 in anyone of lattices, Fisher's exact test was used to calculate exact *p* value. The significant test level was adjusted by Bonferroni multiple test correction. Namely, the level of statistical significance for these pairwise tests was set at 0.05/N, where N was the number of pairwise comparisons for each STR. In addition, univariate odds ratios (ORs) with 95% confidence intervals (CI) of different allele and haplotype frequencies were determined as a measure of the strength of association. When actual frequency (A) in certain lattice was 0, A in four lattices would add 0.5 to calculate OR value. The Y‐STR loci containing alleles with significantly different frequency formed haploid and were compared haplotype frequencies by analysis mentioned above. To adjust the effect of confounding factors, the unconditional logistic regression analysis was further performed to obtain the association between allele genotypes (or haploid haplotypes) and the impulsive aggressive behavior.

## 3 RESULTS

### 3.1 Allele frequencies at 22 Y‐STR loci in offenders and controls

Characteristics of offenders and controls are summarized in Table 2. No significant differences were observed in mean age (*p* = .156), marital status (*p* = .447), educational level (*p* = .124), or geographical region (*p* = .28) between the two groups. For MOAS test, all offenders showed physical aggression against other people (MOAS score >0), with or without verbal aggression and physical aggression against objects. In addition, all recruited offenders lacked expression of physical aggression against self. The total MOAS mean score of the offenders was 21.2. In contrast, because individuals with any aggressive behavior were excluded in the control group, the total MOAS score of the controls was 0.

**Table 2:** Characteristics of recruited participants

Characteristic	Offenders	Controls	*p* value
Age (years)			
Mean (*SD*)	33.8 (10.3)	33.4 (14.3)	.16
Range	13–72	10–80	
Marital status (%)			
Unmarried	19.6	14.5	.45
Married	64.9	73.2	
Divorced	14.5	12.3	
Educational level (%)			
Illiterate	1.1	2.3	.12
Elementary education	12.2	17.6	
Secondary education	84.5	74.7	
Higher education	2.2	5.4	
Geographical region (%)			
North of Changjiang River	54.6	56.3	.28
South of Changjiang River	45.4	43.7	

We first compared allele frequencies of 22 STR loci between offenders and controls by chi‐square test. As shown in Tables 3 and 4 and Table S1–S23, offenders with impulsive aggressive behavior exhibited a significant difference in allele frequencies at two Y‐STR loci (DYS448 and DYS456) (both *p* < .05). In contrast, there were no marked differences in allele frequencies at other 20 Y‐STR loci (All *p* values were more than .05), and thus we did not further compare their single allele frequency between two groups. Further analysis revealed that the frequency of allele 18 at DYS448 was significant lower in the offender group compared to the control group (*p* = .003, OR = 0.57, 95% CI = 0.39–0.82), whereas the frequency of allele 22 at DYS448 was significant higher in the offender group compared to the control group (*p* = .003, OR = 7.45, 95% CI = 1.57–35.35). The frequencies of other alleles at DYS448 showed no statistical significance between the two groups (*p* > .05/6). Similarly, we found that the frequency of allele 17 at DYS456 was significantly higher in the offender group than in the control group (*p* = .006, OR = 0.48, 95% CI = 0.28–0.82), whereas the frequencies of other alleles at DYS456 showed no statistical significance between the two groups.

**Table 3:** Comparisons of allele frequencies of DYS 448 locus between offenders and controls

Alleles	Offenders (*n* = 271)	Controls (*n* = 492)	χ^2^	*p* value	OR	95% CI
17	5 (1.85)	9 (1.83)	0.00	.99	1.01	0.34–3.04
18	48 (17.71)	135 (27.44)	9.07	.003	0.57	0.39–0.82
19	113 (41.70)	169 (34.35)	4.05	.044	1.37	1.01–1.85
20	73 (26.94)	131 (26.63)	0.01	.93	1.02	0.73–1.42
21	22 (8.12)	43 (8.74)	0.09	.77	0.92	0.54–1.58
22	9 (3.32)	3 (0.61)	8.75	.003	7.45	1.57–35.35
χ^2^	16.43					
*p* value	.01					

The number in parentheses indicates frequency (%); allele frequencies with lower than 1% in both groups were removed; the significant test level is set at 0.05/6 ≈ 0.0083.

**Table 4:** Comparisons of allele frequencies of DYS456 locus between offenders and controls

Alleles	Offenders (*n* = 271)	Controls (*n* = 492)	χ^2^	*p* value	OR	95% CI
13	5 (1.85)	12 (2.44)	0.28	.59	0.75	0.26–2.16
14	55 (20.30)	72 (14.63)	4.04	.45	1.49	1.01–2.19
15	141 (52.03)	236 (47.97)	1.15	.28	1.18	0.87–1.58
16	44 (16.24)	88 (17.89)	0.33	.56	0.89	0.60–1.32
17	19 (7.01)	67 (13.62)	7.63	.01	0.48	0.28–0.82
18	5 (1.85)	12 (2.44)	0.28	.59	0.75	0.26–2.16
χ^2^	11.53					
*p* value	.04					

The number in parentheses indicates frequency (%); allele frequencies with lower than 1% in both groups were removed; the significant test level was set at 0.05/6 ≈ 0.0083.

### 3.2 Haploid haplotype frequencies at DYS448‐DYS456 in offenders and controls

As seen in Table 5, there was a significant difference in the frequency of haploid haplotype DYS448‐DYS456 between the two groups (*p *<* *.05). Specifically, the frequency of DYS448‐DYS456‐22‐15 in offenders and controls was 3.32% and 0.41%, respectively, showing a significant difference (*p *<* *.05/18). In contrast, there were no significant differences in the frequencies of other haploid haplotype DYS448‐DYS456 between two groups.

**Table 5:** Comparison of DYS448‐ DYS456 haplotype frequencies between offenders and controls

Haplotype	Offenders (*n* = 271)	Controls (*n* = 492)	χ^2^	*p* value	OR	95% CI
18–14	10 (3.69)	15 (3.05)	0.23	.63	1.22	0.54–2.75
18–15	17 (6.27)	47 (9.55)	2.45	.12	0.63	0.36–1.13
18–16	8 (2.95)	21 (4.27)	0.83	.36	0.68	0.30–1.56
18–17	10 (3.69)	38 (7.72)	4.82	.03	0.46	0.22–0.93
18–18	2 (0.74)	9 (1.83)	1.47	.23	0.40	0.09–1.86
19–14	30 (11.07)	34 (6.91)	3.94	.047	1.68	1.00–2.81
19–15	55 (20.30)	79 (16.06)	2.17	.14	1.33	0.91–1.95
19–16	19 (7.01)	30 (6.10)	0.24	.62	1.16	0.64–2.11
19–17	5 (1.85)	21 (4.27)	3.12	.08	0.42	0.16–1.13
20–13	3 (1.11)	6 (1.22)	0.02	.89	0.91	0.23–3.66
20–14	13 (4.80)	15 (3.05)	1.51	.22	1.60	0.75–3.42
20–15	41 (15.13)	79 (16.06)	0.11	.74	0.93	0.62–1.41
20–16	13 (4.80)	23 (4.67)	0.01	.94	1.03	0.51–2.06
20–17	3 (1.11)	6 (1.22)	0.02	.89	0.91	0.23–3.66
21–14	0 (0.00)	6 (1.22)	3.33	.07	0.26	0.03–2.09
21–15	17 (6.27)	26 (5.28)	0.32	.57	1.20	0.64–2.25
21–16	2 (0.74)	9 (1.83)	1.47	.23	0.40	0.09–1.86
22–15	9 (3.32)	2 (0.41)	10.45	.001	8.42	1.81–39.24
χ^2^	33.43					
*p* value	.010					

The number in parentheses indicates frequency (%); haplotype frequencies with lower than 1% in both groups were removed; the significant test level was set 0.05/18 ≈ 0.0028.

### 3.3 Multivariate logistic regression analysis on influential factors of impulsive aggressive behavior

To adjust the effect of confounding factor, the unconditional logistic regression analysis was performed. Age, marital status, education level, geographic region, DYS448 allele types, DYS456 allele types, and DYS448‐DYS456 haplotypes of participants were regarded as independent variables, while the impulsive aggression was regarded as the dependent variable. The significant associations are shown in Table 6. The results showed that DYS448‐22 allele is a susceptible factor for impulsive aggression (*p* = .02, OR = 4.79, 95% CI = 1.28–17.91), whereas DYS448‐18 allele (*p* = .026, OR = 0.65, 95% CI = 0.44–0.95) and DYS456‐17 allele (*p* = .04, OR = 0.57, 95% CI = 0.33–0.99) are the resistant factors for impulsive aggression. Unfortunately, the DYS448‐DYS456 haplotypes appear to be less related to the impulsive aggression (data not shown).

**Table 6:** Significant association between allele types and impulsive aggression by unconditional logistic regression analysis

Variables	B	*SE*	Wald value	*df*	*p* value	OR (95% CI)
DYS448‐18	−0.43	0.20	10.94	1	.026	0.65 (0.44–0.95)
DYS448‐22	1.57	0.67	5.43	1	.020	4.79 (1.28–17.91)
DYS456‐17	−0.56	0.280	4.04	1	.044	0.57 (0.33–0.99)
Constant	−0.47	0.09	28.27	1	.000	0.63

Allele types (or haloid haplotypes) without significant association with impulsive aggression are not shown. Age, marital status, education level, geographic region, DYS448 allele type, DYS456 allele type, and DYS448‐DYS456 haplotypes of participants were regarded as independent variables, while the aggressive behavior was regarded as the dependent variable.

## 4 DISCUSSION

This is the first study to investigate the association between Y‐STR loci and human male impulsive aggression. By comparing allele and haplotype frequencies of the selected 22 Y‐STRs between male offenders with impulsive aggression and controls, and further association analysis, we found that alleles 18 and 22 at DYS448 and allele 17 at DYS456 are associated with impulsive aggression.

Impulsive aggressive behavior often leads to serious consequences such as crime due to its uncontrollability and lack of rational judgment. Men tend to be more aggressive than women, since androgen is thought to play a key role in the impulsive aggressive behavior (de Almeida, Cabral, & Narvaes, 2015). Aluja, Garcia, Blanch, and Fibla (2011) found that men with less CAG repeats and more GGN repeats in androgen receptor gene was prone to exhibit aggressive behavior, suggesting that there is a correlation between polymorphisms of androgen receptor gene and the aggressive behavior. There is evidence (Carey, 1992; Hesselbrock, 1991) that male‐specific Y chromosome is associated with aggressive behavior. Although many characteristics of the individual are affected by Y chromosome, it is little known about the effects of the related genes on the Y chromosome on the aggressive behavior. Lovell‐Badge (2005) proposed that SRY does not affect directly the aggressive behavior, but exerts its effect in an indirect way. For example, SRY regulates the synthesis of catecholamine hormones by regulating the proximal promoter of TH gene (Zhang et al., 2010); SRY enhances the catalytic activity of MAO‐A, leading to inhibition of the activity of catecholamine hormones (Wu, Chen, Li, Lau, & Shih, 2009).Therefore, Lee proposed (Lee & Harley, 2012) that SRY gene, as a genetic basis for differences in sex‐related responses, is highly related to male impulsive aggressive behavior. In addition, there are a number of genes located on non‐Y chromosome to correlate with aggressive behavior, such as 5‐HT (Retz, Rosler, Supprian, Retz‐Junginger, & Thome, 2003) and DA system candidate genes (Retz, Retz‐Junginger, Supprian, Thome, & Rosler, 2004), nuclear receptor 2E1 (NR2E1) gene (Kumar et al., 2008)in human chromosome 6q21‐22 region, catechol‐o‐methyltransferase (COMT) gene in human chromosome 22q11.2 region(Vevera et al., 2009), monoamine oxidase (MAO) gene in the Xp11‐23~11‐4 region (Manuck, Flory, Ferrell, Mann, & Muldoon, 2000), and estrogen receptor alpha gene (Westberg et al., 2003). Taken together, these findings indicate a significant genetic basis. Thus, it is necessary to expand the research scope of candidate genes for aggression.

Y chromosome is the smallest one in the human genome, accounting for 2%–3% of the entire haploid genome. 95% of Y chromosome genomes are male‐specific, containing 23 million base pairs of male‐specific euchromosome (Skaletsky et al., 2003).Y chromosome genetic analysis plays an important role in the research of human male evolution, complementary medicine, and forensic genetics (Jobling & Tyler‐Smith, 2003). STR generally located within the non‐coding regions of DNA throughout the human genome are repeating DNA sequences that contain 2–6 base pairs (Edwards, Civitello, Hammond, & Caskey, 1991). Genetic polymorphism analysis of STR loci has been widely employed in a variety of fields, such as genetic mapping, positional cloning, paternity tests, linkage analysis of disease mechanisms, tumor biology, population genetics, and evolutionary biology. STR loci on the Y chromosome which are mostly located in the non‐recombinant regions of Y chromosome show a haploid paternal inheritance. Thus, by screening the selected 22 Y‐STR loci, we may found potential Y‐STR loci and haploid haplotypes associated with male impulsive aggression behavior, suggesting an important significance for further mapping of the candidate genes.

In this study, we found that genetic polymorphisms of two Y‐STR loci (DYS448 and DYS456) were associated with impulsive aggressive behavior. Single allele analysis revealed that individuals carrying DYS448‐22 have a risk of 7.45 times for impulsive aggressive behavior as compared to those without the allele. In contrast, individuals carrying DYS448‐18 or DYS456‐17 have a risk of 0.57 times or 0.48 times, respectively, for impulsive aggressive behavior than those lacking the allele. And the multivariate regression analysis confirmed the results. Although the frequency of haploid haplotype 22‐15 on the DYS448‐DYS456 (DYS448‐DYS456‐22‐15) was significantly higher in offenders than in controls, the multivariate regression analysis failed to show that the DYS448‐DYS456‐22‐15 is associated with the aggression. Based on these results, we concluded that DYS448‐22 is susceptible to male impulsive aggressive behavior, whereas DYS456‐17 and DYS448‐18 are resistant to this behavior. Kittles et al. (1999) conducted one study on the association between alcoholism antisocial aggressive behavior and 7 Y‐STR loci(DYS388, DYS389, DYS390, DYS391, DYS392, DYS393, and DYS394)in Finland, but failed to find any association. In their study, less Y‐STR loci were used, without DYS448 and DYS456 leading to our positive results.

Lee and Harley (2012) proposed the following possible mechanisms for male impulsive aggressive behaviors: SRY promotes the release of catecholamine hormones into the blood and further into the peripheral organs and muscles, inhibits monoamine oxidase A in the prefrontal brain, enhances physical activity by boosting the concentration of TH and DA in the substantia nigra, as well as improves the secretion of norepinephrine in the brain locus. Additionally, there is evidence that SRY downregulates the expression of the estrogen receptor, and in turn attenuates the inhibitory effects of estrogen on male impulsive behavior (Tao et al., 2012). DYS448 and DYS456 loci on the Y chromosome may be linked to SRY (Beltramo, Pena, & Lojo, 2015; Gopinath et al., 2013; Liu, Qiao, Li, Yan, & Chen, 2013). However, whether the two loci affects male impulsive aggressive behavior by above mechanisms needs to be further investigated.

In summary, our results suggest that DYS448 and DYS456 loci are associated with male impulsive aggressive behavior. Specifically, DYS448‐22 may be susceptible factors of male impulsive aggressive behavior, whereas DYS448‐18 and DYS456‐17 may be protective factors. Due to the limited number of samples and rare frequency of candidate alleles and haplotypes, we should be careful with our conclusions. Therefore, our findings need to be further confirmed in a larger‐scale study with thousands or ten thousands of samples. Again, it is unclear how these genetic markers affect this behavior. Further research will require a great increase in the sample number, search specific candidate genes on these genetic regions, and further address the potential mechanisms.

## CONFLICT OF INTEREST

The authors claimed that they have no conflict of interest.
