# Supplementary material for: Linking Y‐chromosomal short tandem repeat loci to human male impulsive aggression
Source: Brain Behav. 2017 Oct 16;7(11):e00855. doi: 10.1002/brb3.855 (PMC5698871; doi:10.1002/brb3.855)
Supplement: Supplementary file 1 [file BRB3-7-e00855-s001.docx]

Table S1.Comparisons of allele frequencies of DYS 576 locus between offenders and controls

| Alleles | Offenders (n=271) | Controls(n=492) |
| --- | --- | --- |
| 14 | 2（0.74） | 8（1.63） |
| 15 | 1（0.37） | 5（1.02） |
| 16 | 21（7.75） | 32（6.50） |
| 17 | 59（21.77） | 130（26.42） |
| 18 | 95（35.06） | 158（32.11） |
| 19 | 59（21.77） | 107（21.75） |
| 20 | 26（9.59） | 37（7.52） |
| 21 | 7（2.58） | 12（2.44） |
| 22 | 1（0.37） | 3（0.61） |

Note: the number in parentheses indicates frequency (%); allele frequencies with lower than 1% in both groups were not removed.

Table S1-1.Comparisons of allele frequencies of DYS 576 locus between offenders and controls

| Alleles | Offenders (n=271) | Controls(n=492) |
| --- | --- | --- |
| 14 | 2（0.74） | 8（1.63） |
| 16 | 21（7.75） | 32（6.50） |
| 17 | 21（7.75） | 32（6.50） |
| 18 | 59（21.77） | 130（26.42） |
| 19 | 95（35.06） | 158（32.11） |
| 20 | 59（21.77） | 107（21.75） |
| 21 | 26（9.59） | 37（7.52） |
| χ^2^ | 4.32 | |
| p-value | 0.633 | |

Note: the number in parentheses indicates frequency (%); allele frequencies with lower than 1% in both groups were removed.

Table S2.Comparisons of allele frequencies of DYS389 I locus between offenders and controls

| Alleles | Offenders (n=271) | Controls(n=492) |
| --- | --- | --- |
| 11 | 2（0.74） | 4（0.81） |
| 12 | 141（52.03） | 275（55.89） |
| 13 | 74（27.31） | 127（25.81） |
| 14 | 51（18.82） | 84（17.07） |
| 15 | 1（0.37） | 2（0.41） |
| 22 | 2 ( 0.74） | 0（0.00） |

Note: the number in parentheses indicates frequency (%); allele frequencies with lower than 1% in both groups were not removed.

Table S2-1.Comparisons of allele frequencies of DYS389I locus between offenders and controls

| Alleles | Offenders (n=271) | Controls(n=492) |
| --- | --- | --- |
| 12 | 141（52.03） | 275（55.89） |
| 13 | 74（27.31） | 127（25.81） |
| 14 | 51（18.82） | 84（17.07） |
| χ^2^ | 0.92 | |
| p-value | 0.630 | |

Note: the number in parentheses indicates frequency (%); allele frequencies with lower than 1% in both groups were removed.

Table S3.Comparisons of allele frequencies of DYS 448 locus between offenders and controls

| Alleles | Offenders (n=271) | Controls(n=492) |
| --- | --- | --- |
| 16 | 0（0.00） | 1（0.20） |
| 17 | 5（1.85） | 9（1.83） |
| 18 | 48（17.71） | 135（27.44） |
| 19 | 113（41.70） | 169（34.35） |
| 19.2 | 1（0.37） | 0（0.00） |
| 20 | 73（26.94） | 131（26.63） |
| 20.2 | 0（0.00） | 1（0.20） |
| 21 | 22（8.12） | 43（8.74） |
| 22 | 9（3.32） | 3（0.61） |

Note: the number in parentheses indicates frequency (%); allele frequencies with lower than 1% in both groups were not removed.

Table S4.Comparisons of allele frequencies of DYS38 II locus between offenders and controls

| Alleles | Offenders (n=271) | Controls(n=492) |
| --- | --- | --- |
| 26 | 2（0.74） | 5（1.02） |
| 27 | 27（9.96） | 40（8.13） |
| 28 | 88（32.47） | 150（30.49） |
| 29 | 86（31.73） | 158（32.11） |
| 30 | 52（19.19） | 98（19.92） |
| 31 | 14（5.17） | 30（6.10） |
| 32 | 2（0.74） | 10（2.03） |
| 33 | 0（0.00） | 1（0.20） |

Note: the number in parentheses indicates frequency (%); allele frequencies with lower than 1% in both groups were not removed.

Table S4-1.Comparisons of allele frequencies of DYS38 II locus between offenders and controls

| Alleles | Offenders (n=271) | Controls(n=492) |
| --- | --- | --- |
| 26 | 2（0.74） | 5（1.02） |
| 27 | 27（9.96） | 40（8.13） |
| 28 | 88（32.47） | 150（30.49） |
| 29 | 86（31.73） | 158（32.11） |
| 30 | 52（19.19） | 98（19.92） |
| 31 | 14（5.17） | 30（6.10） |
| 32 | 2（0.74） | 10（2.03） |
| χ^2^ | 3.21 | |
| p-value | 0.782 | |

Note: the number in parentheses indicates frequency (%); allele frequencies with lower than 1% in both groups were removed.

Table S5.Comparisons of allele frequencies of DYS19 locus between offenders and controls

| Alleles | Offenders (n=271) | Controls(n=492) |
| --- | --- | --- |
| 12 | 0（0.00） | 1（0.20） |
| 13 | 16（5.90） | 15（3.05） |
| 14 | 58（21.40） | 98（19.92） |
| 15 | 121（44.65） | 252（51.22） |
| 16 | 57（21.03） | 84（17.07） |
| 17 | 19（7.01） | 42（8.54） |

Note: the number in parentheses indicates frequency (%); allele frequencies with lower than 1% in both groups were not removed.

Table S5-1.Comparisons of allele frequencies of DYS19 locus between offenders and controls

| Alleles | Offenders (n=271) | Controls(n=492) |
| --- | --- | --- |
| 13 | 16（5.90） | 15（3.05） |
| 14 | 58（21.40） | 98（19.92） |
| 15 | 121（44.65） | 252（51.22） |
| 16 | 57（21.03） | 84（17.07） |
| 17 | 19（7.01） | 42（8.54） |
| χ^2^ | 7.22 | |
| p-value | 0.125 | |

Note: the number in parentheses indicates frequency (%); allele frequencies with lower than 1% in both groups were removed.

Table S6.Comparisons of allele frequencies of DYS391 locus between offenders and controls

| Alleles | Offenders (n=271) | Controls(n=492) |
| --- | --- | --- |
| 6 | 1（0.37） | 0（0.00） |
| 9 | 12（4.43） | 17（3.46） |
| 10 | 202（74.54） | 354（71.95） |
| 11 | 55（20.30） | 114（23.17） |
| 12 | 1（0.37） | 6（1.22） |
| 13 | 0（0.00） | 1（0.20） |

Note: the number in parentheses indicates frequency (%); allele frequencies with lower than 1% in both groups were not removed.

Table S6-1.Comparisons of allele frequencies of DYS391 locus between offenders and controls

| Alleles | Offenders (n=271) | Controls(n=492) |
| --- | --- | --- |
| 9 | 12（4.43） | 17（3.46） |
| 10 | 202（74.54） | 354（71.95） |
| 11 | 55（20.30） | 114（23.17） |
| 12 | 1（0.37） | 6（1.22） |
| χ^2^ | 2.63 | |
| p-value | 0.453 | |

Note: the number in parentheses indicates frequency (%); allele frequencies with lower than 1% in both groups were removed.

Table S7.Comparisons of allele frequencies of DYS481 locus between offenders and controls

| Alleles | Offenders (n=271) | Controls(n=492) |
| --- | --- | --- |
| 14 | 0（0.00） | 1（0.20） |
| 19 | 0（0.00） | 2（0.41） |
| 20 | 0（0.00） | 4（0.81） |
| 21 | 14（5.17） | 26（5.28） |
| 22 | 40（14.76） | 78（15.85） |
| 23 | 79（29.15） | 122（24.80） |
| 24 | 64（23.62） | 120（24.39） |
| 25 | 40（14.76） | 90（18.29） |
| 26 | 21（7.75） | 29（5.89） |
| 27 | 4（1.48） | 15（3.05） |
| 28 | 7（2.58） | 4（0.81） |
| 29 | 2（0.74） | 0（0.00） |
| 30 | 0（0.00） | 1（0.20） |

Note: the number in parentheses indicates frequency (%); allele frequencies with lower than 1% in both groups were not removed.

Table S7-1.Comparisons of allele frequencies of DYS481 locus between offenders and controls

| Alleles | Offenders (n=271) | Controls(n=492) |
| --- | --- | --- |
| 21 | 14（5.17） | 26（5.28） |
| 22 | 40（14.76） | 78（15.85） |
| 23 | 79（29.15） | 122（24.80） |
| 24 | 64（23.62） | 120（24.39） |
| 25 | 40（14.76） | 90（18.29） |
| 26 | 21（7.75） | 29（5.89） |
| 27 | 4（1.48） | 15（3.05） |
| 28 | 7（2.58） | 4（0.81） |
| χ^2^ | 11.37 | |
| p-value | 0.123 | |

Note: the number in parentheses indicates frequency (%); allele frequencies with lower than 1% in both groups were removed.

Table S8.Comparisons of allele frequencies of DYS549 locus between offenders and controls

| Alleles | Offenders (n=271) | Controls(n=492) |
| --- | --- | --- |
| 10 | 1（0.37） | 3（0.61） |
| 11 | 20（7.38） | 28（5.69） |
| 12 | 145（53.51） | 279（56.71） |
| 13 | 83（30.63） | 137（27.85） |
| 14 | 18（6.64） | 41（8.33） |
| 15 | 4（1.48） | 4（0.81） |

Note: the number in parentheses indicates frequency (%); allele frequencies with lower than 1% in both groups were not removed.

Table S8-1.Comparisons of allele frequencies of DYS549 locus between offenders and controls

| Alleles | Offenders (n=271) | Controls(n=492) |
| --- | --- | --- |
| 11 | 20（7.38） | 28（5.69） |
| 12 | 145（53.51） | 279（56.71） |
| 13 | 83（30.63） | 137（27.85） |
| 14 | 18（6.64） | 41（8.33） |
| 15 | 4（1.48） | 4（0.81） |
| χ^2^ | 2.96 | |
| p-value | 0.565 | |

Note: the number in parentheses indicates frequency (%); allele frequencies with lower than 1% in both groups were removed.

Table S9.Comparisons of allele frequencies of DYS533 locus between offenders and controls

| Alleles | Offenders (n=271) | Controls(n=492) |
| --- | --- | --- |
| 9 | 0（0.00） | 2（0.41） |
| 10 | 16（5.90） | 41（8.33） |
| 11 | 151（55.72） | 294（59.76） |
| 12 | 86（31.73） | 131（26.63） |
| 13 | 13（4.80） | 23（4.67） |
| 14 | 5（1.85） | 1（0.20） |

Note: the number in parentheses indicates frequency (%); allele frequencies with lower than 1% in both groups were not removed.

Table S9-1.Comparisons of allele frequencies of DYS533 locus between offenders and controls

| Alleles | Offenders (n=271) | Controls(n=492) |
| --- | --- | --- |
| 10 | 16（5.90） | 41（8.33） |
| 11 | 151（55.72） | 294（59.76） |
| 12 | 86（31.73） | 131（26.63） |
| 13 | 13（4.80） | 23（4.67） |
| 14 | 5（1.85） | 1（0.20） |
| χ^2^ | 0.45 | |
| p-value | 0.051 | |

Note: the number in parentheses indicates frequency (%); allele frequencies with lower than 1% in both groups were removed.

Table S10.Comparisons of allele frequencies of DYS438 locus between offenders and controls

| Alleles | Offenders (n=271) | Controls(n=492) |
| --- | --- | --- |
| 8 | 0（0.00） | 1（0.20） |
| 9 | 2（0.74） | 6（1.22） |
| 10 | 213（78.60） | 377（76.63） |
| 11 | 48（17.71） | 90（18.29） |
| 12 | 7（2.58） | 13（2.64） |
| 13 | 1（0.37） | 5（1.02） |

Note: the number in parentheses indicates frequency (%); allele frequencies with lower than 1% in both groups were not removed.

Table S10-1.Comparisons of allele frequencies of DYS438 locus between offenders and controls

| Alleles | Offenders (n=271) | Controls(n=492) |
| --- | --- | --- |
| 9 | 2（0.74） | 6（1.22） |
| 10 | 213（78.60） | 377（76.63） |
| 11 | 48（17.71） | 90（18.29） |
| 12 | 7（2.58） | 13（2.64） |
| 13 | 1（0.37） | 5（1.02） |
| χ^2^ | 1.44 | |
| p-value | 0.837 | |

Note: the number in parentheses indicates frequency (%); allele frequencies with lower than 1% in both groups were removed.

Table S11.Comparisons of allele frequencies of DYS437 locus between offenders and controls

| Alleles | Offenders (n=271) | Controls(n=492) |
| --- | --- | --- |
| 11 | 1（0.37） | 0（0.00） |
| 13 | 4（1.48） | 6（1.22） |
| 14 | 160（59.04） | 290（58.94） |
| 15 | 100（36.90） | 187（38.01） |
| 16 | 6（2.21） | 9（1.83） |

Note: the number in parentheses indicates frequency (%); allele frequencies with lower than 1% in both groups were not removed.

Table S11-1.Comparisons of allele frequencies of DYS437 locus between offenders and controls

| Alleles | Offenders (n=271) | Controls(n=492) |
| --- | --- | --- |
| 13 | 4（1.48） | 6（1.22） |
| 14 | 160（59.04） | 290（58.94） |
| 15 | 100（36.90） | 187（38.01） |
| 16 | 6（2.21） | 9（1.83） |
| χ^2^ | 0.26 | |
| p-value | 0.965 | |

Note: the number in parentheses indicates frequency (%); allele frequencies with lower than 1% in both groups were removed.

Table S12.Comparisons of allele frequencies of DYS570 locus between offenders and controls

| Alleles | Offenders (n=271) | Controls(n=492) |
| --- | --- | --- |
| 10 | 0（0.00） | 1（0.20） |
| 11 | 0（0.00） | 1（0.20） |
| 12 | 0（0.00） | 1（0.20） |
| 14 | 7（2.58） | 5（1.02） |
| 15 | 4（1.48） | 13（2.64） |
| 16 | 38（14.02） | 65（13.21） |
| 17 | 60（22.14） | 120（24.39） |
| 18 | 78（28.78） | 133（27.03） |
| 19 | 53（19.56） | 88（17.89） |
| 20 | 23（8.49） | 51（10.37） |
| 21 | 8（2.95） | 10（2.03） |
| 22 | 0（0.00） | 2（0.41） |
| 23 | 0（0.00） | 1（0.20） |
| 24 | 0（0.00） | 1（0.20） |

Note: the number in parentheses indicates frequency (%); allele frequencies with lower than 1% in both groups were not removed.

Table S12-1.Comparisons of allele frequencies of DYS570 locus between offenders and controls

| Alleles | Offenders (n=271) | Controls(n=492) |
| --- | --- | --- |
| 14 | 7（2.58） | 5（1.02） |
| 15 | 4（1.48） | 13（2.64） |
| 16 | 38（14.02） | 65（13.21） |
| 17 | 60（22.14） | 120（24.39） |
| 18 | 78（28.78） | 133（27.03） |
| 19 | 53（19.56） | 88（17.89） |
| 20 | 23（8.49） | 51（10.37） |
| 21 | 8（2.95） | 10（2.03） |
| χ^2^ | 5.91 | |
| p-value | 0.550 | |

Note: the number in parentheses indicates frequency (%); allele frequencies with lower than 1% in both groups were removed.

Table S13.Comparisons of allele frequencies of DYS635 locus between offenders and controls

| Alleles | Offenders (n=271) | Controls(n=492) |
| --- | --- | --- |
| 18 | 1（0.37） | 0（0.00） |
| 19 | 35（12.92） | 82（16.67） |
| 20 | 71（26.20） | 112（22.76） |
| 21 | 85（31.37） | 151（30.69） |
| 22 | 44（16.24） | 85（17.28） |
| 23 | 24（8.86） | 44（8.94） |
| 24 | 9（3.32） | 14（2.85） |
| 25 | 2（0.74） | 4（0.81） |

Note: the number in parentheses indicates frequency (%); allele frequencies with lower than 1% in both groups were not removed.

Table S13-1.Comparisons of allele frequencies of DYS635 locus between offenders and controls

| Alleles | Offenders (n=271) | Controls(n=492) |
| --- | --- | --- |
| 19 | 35（12.92） | 82（16.67） |
| 20 | 71（26.20） | 112（22.76） |
| 21 | 85（31.37） | 151（30.69） |
| 22 | 44（16.24） | 85（17.28） |
| 23 | 24（8.86） | 44（8.94） |
| 24 | 9（3.32） | 14（2.85） |
| χ^2^ | 2.73 | |
| p-value | 0.741 | |

Note: the number in parentheses indicates frequency (%); allele frequencies with lower than 1% in both groups were removed.

Table S14.Comparisons of allele frequencies of DYS390 locus between offenders and controls

| Alleles | Offenders (n=271) | Controls(n=492) |
| --- | --- | --- |
| 19 | 0（0.00） | 1（0.20） |
| 20 | 1（0.37） | 0（0.00） |
| 21 | 2（0.74） | 4（0.81） |
| 22 | 16（5.90） | 30（6.10） |
| 23 | 116（42.80） | 234（47.56） |
| 24 | 82（30.26） | 146（29.67） |
| 25 | 50（18.45） | 73（14.84） |
| 26 | 3（1.11） | 4（0.81） |
| 28 | 1（0.37） | 0（0.00） |

Note: the number in parentheses indicates frequency (%); allele frequencies with lower than 1% in both groups were not removed.

Table S14-1.Comparisons of allele frequencies of DYS390 locus between offenders and controls

| Alleles | Offenders (n=271) | Controls(n=492) |
| --- | --- | --- |
| 22 | 16（5.90） | 30（6.10） |
| 23 | 116（42.80） | 234（47.56） |
| 24 | 82（30.26） | 146（29.67） |
| 25 | 50（18.45） | 73（14.84） |
| 26 | 3（1.11） | 4（0.81） |
| χ^2^ | 2.47 | |
| p-value | 0.650 | |

Note: the number in parentheses indicates frequency (%); allele frequencies with lower than 1% in both groups were removed.

Table S15.Comparisons of allele frequencies of DYS439 locus between offenders and controls

| Alleles | Offenders (n=271) | Controls(n=492) |
| --- | --- | --- |
| 10 | 17（6.27） | 22（4.47） |
| 11 | 104（38.38） | 199（40.45） |
| 12 | 103（38.01） | 206（41.87） |
| 13 | 43（15.87） | 51（10.37） |
| 14 | 4（1.48） | 11（2.24） |
| 15 | 0（0.00） | 3（0.61） |

Note: the number in parentheses indicates frequency (%); allele frequencies with lower than 1% in both groups were not removed.

Table S15-1.Comparisons of allele frequencies of DYS439 locus between offenders and controls

| Alleles | Offenders (n=271) | Controls(n=492) |
| --- | --- | --- |
| 10 | 17（6.27） | 22（4.47） |
| 11 | 104（38.38） | 199（40.45） |
| 12 | 103（38.01） | 206（41.87） |
| 13 | 43（15.87） | 51（10.37） |
| 14 | 4（1.48） | 11（2.24） |
| χ^2^ | 6.73 | |
| p-value | 0.151 | |

Note: the number in parentheses indicates frequency (%); allele frequencies with lower than 1% in both groups were removed.

Table S16.Comparisons of allele frequencies of DYS392 locus between offenders and controls

| Alleles | Offenders (n=271) | Controls(n=492) |
| --- | --- | --- |
| 10 | 4（1.48） | 3（0.61） |
| 11 | 28（10.33） | 46（9.35） |
| 12 | 38（14.02） | 94（19.11） |
| 13 | 97（35.79） | 174（35.37） |
| 14 | 94（34.69） | 147（29.88） |
| 15 | 10（3.69） | 25（5.08） |
| 16 | 0（0.00） | 3（0.61） |

Note: the number in parentheses indicates frequency (%); allele frequencies with lower than 1% in both groups were not removed.

Table S16-1.Comparisons of allele frequencies of DYS392 locus between offenders and controls

| Alleles | Offenders (n=271) | Controls(n=492) |
| --- | --- | --- |
| 10 | 4（1.48） | 3（0.61） |
| 11 | 28（10.33） | 46（9.35） |
| 12 | 38（14.02） | 94（19.11） |
| 13 | 97（35.79） | 174（35.37） |
| 14 | 94（34.69） | 147（29.88） |
| 15 | 10（3.69） | 25（5.08） |
| χ^2^ | 6.22 | |
| p-value | 0.285 | |

Note: the number in parentheses indicates frequency (%); allele frequencies with lower than 1% in both groups were removed.

Table S17.Comparisons of allele frequencies of DYS643 locus between offenders and controls

| Alleles | Offenders (n=271) | Controls(n=492) |
| --- | --- | --- |
| 7 | 0（0.00） | 1（0.20） |
| 8 | 5（1.85） | 13（2.64） |
| 9 | 28（10.33） | 36（7.32） |
| 10 | 72（26.57） | 122（24.80） |
| 11 | 125（46.13） | 237（48.17） |
| 12 | 34（12.55） | 75（15.24） |
| 13 | 6（2.21） | 7（1.42） |
| 14 | 1（0.37） | 1（0.20） |

Note: the number in parentheses indicates frequency (%); allele frequencies with lower than 1% in both groups were not removed.

Table S17-1.Comparisons of allele frequencies of DYS643 locus between offenders and controls

| Alleles | Offenders (n=271) | Controls(n=492) |
| --- | --- | --- |
| 8 | 5（1.85） | 13（2.64） |
| 9 | 28（10.33） | 36（7.32） |
| 10 | 72（26.57） | 122（24.80） |
| 11 | 125（46.13） | 237（48.17） |
| 12 | 34（12.55） | 75（15.24） |
| 13 | 6（2.21） | 7（1.42） |
| χ^2^ | 4.27 | |
| p-value | 0.512 | |

Note: the number in parentheses indicates frequency (%); allele frequencies with lower than 1% in both groups were removed.

Table S18.Comparisons of allele frequencies of DYS393 locus between offenders and controls

| Alleles | Offenders (n=271) | Controls(n=492) |
| --- | --- | --- |
| 11 | 2（0.74） | 5（1.02） |
| 12 | 138（50.92） | 246（50.00） |
| 13 | 82（30.26） | 145（29.47） |
| 14 | 30（11.07） | 69（14.02） |
| 15 | 19（7.01） | 26（5.28） |
| 16 | 0（0.00） | 1（0.20） |

Note: the number in parentheses indicates frequency (%); allele frequencies with lower than 1% in both groups were not removed.

Table S18-1.Comparisons of allele frequencies of DYS393 locus between offenders and controls

| Alleles | Offenders (n=271) | Controls(n=492) |
| --- | --- | --- |
| 11 | 2（0.74） | 5（1.02） |
| 12 | 138（50.92） | 246（50.00） |
| 13 | 82（30.26） | 145（29.47） |
| 14 | 30（11.07） | 69（14.02） |
| 15 | 19（7.01） | 26（5.28） |
| χ^2^ | 2.27 | |
| p-value | 0.686 | |

Note: the number in parentheses indicates frequency (%); allele frequencies with lower than 1% in both groups were removed.

Table S19.Comparisons of allele frequencies of DYS458 locus between offenders and controls

| Alleles | Offenders (n=271) | Controls(n=492) |
| --- | --- | --- |
| 13 | 2（0.74） | 4（0.81） |
| 14 | 8（2.95） | 15（3.05） |
| 15 | 48（17.71） | 100（20.33） |
| 16 | 45（16.61） | 85（17.28） |
| 17 | 75（27.68） | 121（24.59） |
| 18 | 58（21.40） | 95（19.31） |
| 19 | 19（7.01） | 45（9.15） |
| 20 | 7（2.58） | 24（4.88） |
| 21 | 5（1.85） | 0（0.00） |
| 22 | 2（0.74） | 3（0.61） |
| 23 | 2（0.74） | 0（0.00） |

Note: the number in parentheses indicates frequency (%); allele frequencies with lower than 1% in both groups were not removed.

Table S19-1.Comparisons of allele frequencies of DYS458 locus between offenders and controls

| Alleles | Offenders (n=271) | Controls(n=492) |
| --- | --- | --- |
| 14 | 8（2.95） | 15（3.05） |
| 15 | 48（17.71） | 100（20.33） |
| 16 | 45（16.61） | 85（17.28） |
| 17 | 75（27.68） | 121（24.59） |
| 18 | 58（21.40） | 95（19.31） |
| 19 | 19（7.01） | 45（9.15） |
| 20 | 7（2.58） | 24（4.88） |
| 21 | 5（1.85） | 0（0.00） |
| χ^2^ | 14.01 | |
| p-value | 0.051 | |

Note: the number in parentheses indicates frequency (%); allele frequencies with lower than 1% in both groups were removed.

Table S20.Comparisons of allele frequencies of DYS385 locus between offenders and controls

| Alleles | Offenders (n=271) | Controls(n=492) |
| --- | --- | --- |
| 8-11 | 1（0.37） | 0（0.00） |
| 10-11 | 1（0.37） | 2（0.41） |
| 10-12 | 2（0.74） | 1（0.20） |
| 10-13 | 1（0.37） | 3（0.61） |
| 10-14 | 0（0.00） | 2（0.41） |
| 10-17 | 1（0.37） | 2（0.41） |
| 10-18 | 0（0.00） | 5（1.02） |
| 10-19 | 1（0.37） | 1（0.20） |
| 10-22 | 0（0.00） | 1（0.20） |
| 11-11 | 10（3.69） | 5（1.02） |
| 11-12 | 13（4.80） | 21（4.27） |
| 11-13 | 0（0.00） | 5（1.02） |
| 11-14 | 2（0.74） | 2（0.41） |
| 11-15 | 1（0.37） | 1（0.20） |
| 11-16 | 3（1.11） | 8（1.63） |
| 11-17 | 4（1.48） | 11（2.24） |
| 11-18 | 8（2.95） | 18（3.66） |
| 11-19 | 10（3.69） | 11（2.24） |
| 11-20 | 1（0.37） | 5（1.02） |
| 11-21 | 0（0.00） | 2（0.41） |
| 11-16.2 | 1（0.37） | 0（0.00） |
| 12-12 | 9（3.32） | 16（3.25） |
| 12-13 | 7（2.58） | 7（1.42） |
| 12-14 | 0（0.00） | 6（1.22） |
| 12-15 | 1（0.37） | 4（0.81） |
| 12-16 | 7（2.58） | 22（4.47） |
| 12-16.2 | 0（0.00） | 1（0.20） |
| 12-17 | 17（6.27） | 32（6.50） |
| 12-18 | 12（4.43） | 17（3.46） |
| 12-19 | 14（5.17） | 21（4.27） |
| 12-20 | 9（3.32） | 9（1.83） |
| 12-21 | 3（1.11） | 2（0.41） |
| 12-23 | 0（0.00） | 1（0.20） |
| 13-13 | 17（6.27） | 56（11.38） |
| 13-14 | 11（4.06） | 21（4.27） |
| 13-15 | 1（0.37） | 1（0.20） |
| 13-16 | 2（0.74） | 12（2.44） |
| 13-17 | 7（2.58） | 11（2.24） |
| 13-17.2 | 0（0.00） | 1（0.20） |
| 13-18 | 15（5.54） | 16（3.25） |
| 13-19 | 17（6.27） | 31（6.30） |
| 13-20 | 10（3.69） | 16（3.25） |
| 13-21 | 4（1.48） | 5（1.02） |
| 13-22 | 0（0.00） | 3（0.61） |
| 13-23 | 0（0.00） | 2（0.41） |
| 13-25 | 0（0.00） | 2（0.41） |
| 13-26 | 1（0.37） | 0（0.00） |
| 14-14 | 0（0.00） | 3（0.61） |
| 14-15 | 2（0.74） | 0（0.00） |
| 14-16 | 0（0.00） | 1（0.20） |
| 14-17 | 5（1.85） | 6（1.22） |
| 14-18 | 8（2.95） | 16（3.25） |
| 14-19 | 5（1.85） | 6（1.22） |
| 14-20 | 4（1.48） | 5（1.02） |
| 14-21 | 2（0.74） | 1（0.20） |
| 14-22 | 1（0.37） | 0（0.00） |
| 15-15 | 1（0.37） | 4（0.81） |
| 15-16 | 3（1.11） | 0（0.00） |
| 15-17 | 1（0.37） | 2（0.41） |
| 15-18 | 0（0.00） | 4（0.81） |
| 15-19 | 4（1.48） | 3（0.61） |
| 15-20 | 0（0.00） | 4（0.81） |
| 15-21 | 2（0.74） | 4（0.81） |
| 15-22 | 2（0.74） | 1（0.20） |
| 15-23 | 0（0.00） | 2（0.41） |
| 15-24 | 0（0.00） | 1（0.20） |
| 16-16 | 3（1.11） | 1（0.20） |
| 16-17 | 0（0.00） | 4（0.81） |
| 16-20 | 1（0.37） | 0（0.00） |
| 17-17 | 0（0.00） | 2（0.41） |
| 17-19 | 2（0.74） | 0（0.00） |
| 19-19 | 1（0.37） | 0（0.00） |
| 20-21 | 0（0.00） | 1（0.20） |

Note: the number in parentheses indicates frequency (%); allele frequencies with lower than 1% in both groups were not removed.

Table S20-1.Comparisons of allele frequencies of DYS385 locus between offenders and controls

| Alleles | Offenders (n=271) | Controls(n=492) |
| --- | --- | --- |
| 10-18 | 0（0.00） | 5（1.02） |
| 11-11 | 10（3.69） | 5（1.02） |
| 11-12 | 13（4.80） | 21（4.27） |
| 11-13 | 0（0.00） | 5（1.02） |
| 11-16 | 3（1.11） | 8（1.63） |
| 11-17 | 4（1.48） | 11（2.24） |
| 11-18 | 8（2.95） | 18（3.66） |
| 11-19 | 10（3.69） | 11（2.24） |
| 11-20 | 1（0.37） | 5（1.02） |
| 12-12 | 9（3.32） | 16（3.25） |
| 12-13 | 7（2.58） | 7（1.42） |
| 12-14 | 0（0.00） | 6（1.22） |
| 12-16 | 7（2.58） | 22（4.47） |
| 12-17 | 17（6.27） | 32（6.50） |
| 12-18 | 12（4.43） | 17（3.46） |
| 12-19 | 14（5.17） | 21（4.27） |
| 12-20 | 9（3.32） | 9（1.83） |
| 12-21 | 3（1.11） | 2（0.41） |
| 13-13 | 17（6.27） | 56（11.38） |
| 13-14 | 11（4.06） | 21（4.27） |
| 13-16 | 2（0.74） | 12（2.44） |
| 13-17 | 7（2.58） | 11（2.24） |
| 13-18 | 15（5.54） | 16（3.25） |
| 13-19 | 17（6.27） | 31（6.30） |
| 13-20 | 10（3.69） | 16（3.25） |
| 13-21 | 4（1.48） | 5（1.02） |
| 14-17 | 5（1.85） | 6（1.22） |
| 14-18 | 8（2.95） | 16（3.25） |
| 14-19 | 5（1.85） | 6（1.22） |
| 14-20 | 4（1.48） | 5（1.02） |
| 15-16 | 3（1.11） | 0（0.00） |
| 15-19 | 4（1.48） | 3（0.61） |
| 16-16 | 3（1.11） | 1（0.20） |
| χ^2^ | 45.9 | |
| p-value | 0.053 | |

Note: the number in parentheses indicates frequency (%); allele frequencies with lower than 1% in both groups were removed.

Table S21.Comparisons of allele frequencies of DYS456 locus between offenders and controls

| Alleles | Offenders (n=271) | Controls(n=492) |
| --- | --- | --- |
| 13 | 5（1.85） | 12（2.44） |
| 14 | 55（20.30） | 72（14.63） |
| 15 | 141（52.03） | 236（47.97） |
| 16 | 44（16.24） | 88（17.89） |
| 17 | 19（7.01） | 67（13.62） |
| 18 | 5（1.85） | 12（2.44） |
| 19 | 2（0.74） | 3（0.61） |
| 20 | 0（0.00） | 1（0.20） |
| 23 | 0（0.00） | 1（0.20） |

Note: the number in parentheses indicates frequency (%); allele frequencies with lower than 1% in both groups were not removed.

Table S22.Comparisons of allele frequencies of DY_GATA_H4 locus between offenders and controls

| Alleles | Offenders (n=271) | Controls(n=492) |
| --- | --- | --- |
| 9 | 0（0.00） | 1（0.20） |
| 10 | 13（4.80） | 21（4.27） |
| 11 | 92（33.95） | 166（33.74） |
| 12 | 141（52.03） | 255（51.83） |
| 13 | 24（8.86） | 44（8.94） |
| 14 | 1（0.37） | 5（1.02） |

Note: the number in parentheses indicates frequency (%); allele frequencies with lower than 1% in both groups were not removed.

Table S22-1.Comparisons of allele frequencies of DY_GATA_H4 locus between offenders and controls

| Alleles | Offenders (n=271) | Controls(n=492) |
| --- | --- | --- |
| 10 | 13（4.80） | 21（4.27） |
| 11 | 92（33.95） | 166（33.74） |
| 12 | 141（52.03） | 255（51.83） |
| 13 | 24（8.86） | 44（8.94） |
| 14 | 1（0.37） | 5（1.02） |
| χ^2^ | 1.04 | |
| p-value | 0.903 | |

Note: the number in parentheses indicates frequency (%); allele frequencies with lower than 1% in both groups were removed.

Table S23.Comparison of DYS448- DYS456 haplotype frequencies between offenders and controls

| Haplotype | Offenders (n=271) | Controls(n=492) |
| --- | --- | --- |
| 16-16 | 0（0.00） | 1（0.20） |
| 17-14 | 1（0.37） | 1（0.20） |
| 17-15 | 2（0.74） | 3（0.61） |
| 17-16 | 2（0.74） | 3（0.61） |
| 17-17 | 0（0.00） | 2（0.41） |
| 18-13 | 0（0.00） | 1（0.20） |
| 18-14 | 10（3.69） | 15（3.05） |
| 18-15 | 17（6.27） | 47（9.55） |
| 18-16 | 8（2.95） | 21（4.27） |
| 18-17 | 10（3.69） | 38（7.72） |
| 18-18 | 2（0.74） | 9（1.83） |
| 18-19 | 1（0.37） | 3（0.61） |
| 18-20 | 0（0.00） | 1（0.20） |
| 19-13 | 1（0.37） | 3（0.61） |
| 19-14 | 30（11.07） | 34（6.91） |
| 19-15 | 56（20.66） | 79（16.06） |
| 19-16 | 19（7.01） | 30（6.10） |
| 19-17 | 5（1.85） | 21（4.27） |
| 19-18 | 2（0.74） | 2（0.41） |
| 19-19 | 1（0.37） | 0（0.00） |
| 19.2-14 | 1（0.37） | 0（0.00） |
| 20-13 | 3（1.11） | 6（1.22） |
| 20-14 | 13（4.80） | 15（3.05） |
| 20-15 | 41（15.13） | 79（16.06） |
| 20-16 | 13（4.80） | 23（4.67） |
| 20-17 | 3（1.11） | 6（1.22） |
| 20-18 | 0（0.00） | 1（0.20） |
| 20-23 | 0（0.00） | 1（0.20） |
| 20.2-14 | 0（0.00） | 1（0.20） |
| 21-13 | 1（0.37） | 2（0.41） |
| 21-14 | 0（0.00） | 6（1.22） |
| 21-15 | 17（6.27） | 26（5.28） |
| 21-16 | 2（0.74） | 9（1.83） |
| 21-17 | 1（0.37） | 0（0.00） |
| 21-18 | 1（0.37） | 0（0.00） |
| 22-15 | 8（2.95） | 2（0.41） |
| 22-16 | 0（0.00） | 1（0.20） |

Note: the number in parentheses indicates frequency (%); Haplotype frequencies with lower than 1% in both groups were not removed.
